# Tailoring charge transport in perovskite photovoltaics *via* chalcogen-thiophene molecular bridges

**DOI:** 10.1039/d6ra00231e

**Published:** 2026-03-30

**Authors:** Mustafa Kareem, Ethar Yahya Salih, M. M. Rekha, Anjan Kumar, Vatsal Jain, Chandra Kant Bhardwaj

**Affiliations:** a College of Remote Sensing and Geophysics, Al-Karkh University of Science Al-Karkh Side, Haifa St. Hamada Palace Baghdad 10011 Iraq dr.mustafa@kus.edu.iq; b College of Science, University of Warith Al-Anbiyaa 56001 Karbala Iraq; c College of Energy and Environmental Sciences, Al-Karkh University of Science Baghdad 10081 Iraq; d Department of Chemistry and Biochemistry, School of Sciences, JAIN (Deemed to be University) Bangalore Karnataka India; e Department of Electronics and Communication Engineering, GLA University Mathura-281406 India; f Centre for Research Impact & Outcome, Chitkara University Institute of Engineering and Technology, Chitkara University Rajpura 140401 Punjab India; g Department of Chemistry, Graphic Era Hill University Dehradun India; h Centre for Promotion of Research, Graphic Era Deemed to be University Dehradun Uttarakhand-248002 India

## Abstract

We present in this research the theoretical design and development of a Lewis base molecule, *n*-Bu4S, formed of fused thiophene units bridged by tetra-pyridine, as a versatile interfacial passivation layer for perovskite solar cells (PSCs). Strong host–guest interactions between *n*-Bu4S and under-coordinated Pb^2+^ essentially reduce interfacial recombination, hence improving charge extraction and device stability. Then, a typical structure based on ITO/SnO_2_/perovskite/*n*-Bu4S/Spiro-OMeTAD/Au PSCs is proposed and simulated. Using SCAPS-1D numerical simulations shows that adding *n*-Bu4S greatly enhances the built-in potential, charge carrier kinetics, and overall device performance. The best-performing devices attained a simulated power conversion efficiency (PCE) of 24%. Furthermore, device-level investigations displayed the important effect of adjusting parameters such as perovskite thickness, defect density, carrier mobility, and shallow acceptor concentration. The simulated Nyquist analysis confirmed enhanced recombination resistance for the *n*-Bu4S-treated device. The results underline the need for interfacial and bulk engineering to obtain efficient and thermally stable PSCs, hence positioning *n*-Bu4S as a potential method for next-generation perovskite photovoltaics.

## Abstract 2

This article reports the design and development of a Lewis base molecule, *n*-Bu4S, formed of fused thiophene units bridged by tetra-pyridine, as a versatile interfacial passivation layer for PSCs.

## Graphical abstract

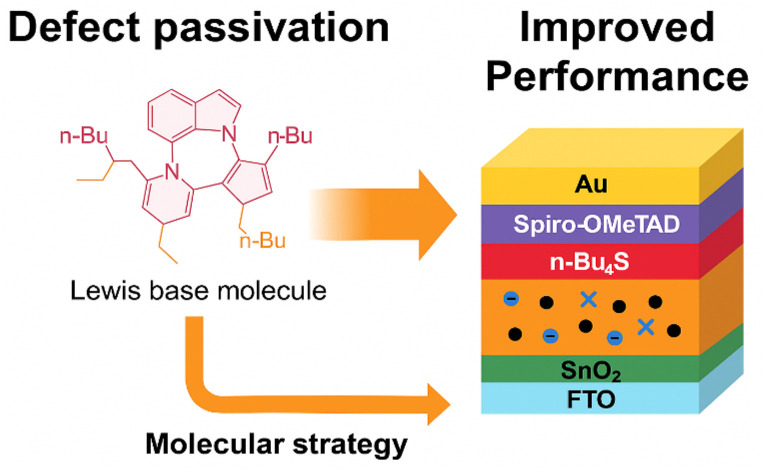

## 1. Introduction

Currently, photovoltaics (PVs) as a power source are becoming economically competitive with conventional energy technologies. Renewable energy technologies translate sources of clean energy, such as solar radiation or wind, into usable power sources like electricity without releasing CO_2_ during this conversion process.^1,2^ Metal halide perovskite materials have achieved significant advancements in optoelectronics owing to their widely adjustable compositions obtained through facile processing methods, as well as their exceptional light absorption, low exciton binding energy, increased charge mobility, and a prolonged carrier diffusion length.^3,4^ Perovskite solar cells (PSCs) have developed significantly during the last decade, currently with a verified PCE of 27%.^5^ Nevertheless, the interfaces between perovskite and charge-transporting layers still suffer from a high level of carrier traps and dangling bonds.^6^ Passivating the perovskite top and buried interfaces and suppressing nonradiative recombination with organic spacers are efficient methods to enhance PSC performance.^7,8^

Interfacial engineering has been extensively investigated to improve charge transfer and interfacial interactions in diverse optoelectronics.^9,10^ To control the crystallization process, molecular passivators, including N, O, or S atoms with lone pair electrons, have been shown to modify perovskite grain formation efficiently and reduce traps *via* Lewis acid-base coupling.^11,12^ For example, carbohydrazide,^13^ phenethylammonium,^14^ hydantoin 15, dimethyl sulfide,^16^ and others have been reported to develop Lewis adducts with under-coordinated Pb^2+^ ions in the perovskite, reducing nucleation centers and retarding crystallization, resulting in larger grains and reduced defect density. For more chemically specified passivation, chalcogenide-based functional ligands have emerged as key molecules owing to their strong binding affinity to deep-level Pb^2+^ defects and halide vacancies, strongly mitigating trap states.^17–19^ Recently, Lammar *et al.* reported a benzothieno[3,2-*b*]benzothiophene (BTBT) interlayer for passivating the interface between the hole transport layer (HTL) and the perovskite in PSCs. The passivation of the HTL/perovskite interface through the BTBT reduces a (redox) reaction and improves the open-circuit voltage (*V*_OC_) due to minimizing the non-radiative recombination. The BTBT-based PSC realized an improved PCE of 18.6% with better ambient stability.^20^ Following this, Sadhu *et al.* used three types of chalcogen interlayers, namely triphenylphosphine oxide (TPPO), triphenylphosphine sulfide (TPPS), and triphenylphosphine selenide (TPPSe), to passivate defects in the PSCs. The three interface passivators enhanced the PCE of the PSCs in comparison with the reference device, recording 15.19% PCE with a low hysteresis index. Besides, TPPS and TPPSe passivation exhibit higher stability because of their greater binding with Pb^2+^ and Pb^0^ sites. This allows them to eliminate a wider variety of traps and retain dative interactions even under environmental stressors.^21^ Finkenauer *et al.* introduced a combination of amine and chalcogenide ligands to develop crystalline and oriented *α*-FAPbI_3_ films by the sequential deposition method. The combined additives tuned the perovskite intermediate phase and controlled the crystal growth, resulting in perovskites with suppressed defect densities and boosted charge carrier lifetimes.^22^ In 2025, Azam *et al.* synthesized two chalcogen-thiophene Lewis bases possessing tetra-pyridine as a bridge to passivate defects in mixed-cation PSC. The Pb^2+^ and I^−^ vacancy defects interacted with chalcogen and pyridine groups through the production of the Lewis acid-base adduct, leading to reduction carrier recombination. This treatment realized a certified PCE of 25.18% for PSCs.^23^

Inspired by the above discussion, we develop a molecular bridging strategy by designing a chalcogen-pyridine compound, named 6,6′-bis(2,3,5,6,9,10 hexabutoxydithieno[2′,3′,4′,5′:4,5; 2″,3″,4″,5″:8,9]triphenyleno[1,12-*bcd*]pyridin-8-yl)-2,2′-bipyridine (*n*-Bu4S), to passivate buried defects at the perovskite/HTL interface of n-i-p-structured mixed-cation PSCs. We show that the incorporation of an *n*-Bu4S interlayer leads to a positive influence on the HTL/perovskite interface and solar cell characteristics. The simulated external quantum efficiency (EQE) calculations exhibit that the *n*-Bu4S passivator results in an improved charge carrier transport across the perovskite/HTL contact and consequently gives rise to a higher short-circuit current density (*J*_SC_). Our results demonstrate that the cell modified with *n*-Bu4S achieves a theoretical PV performance of 24%, together with an enhanced fill factor (FF) of 85.07% and *V*_OC_ of 1.4 V. Additionally, the proposed device also demonstrated enhanced thermal stability.

## 2. Methodology

SCAPS-1D, a program created by the University of Gent in Belgium, was used to carry out the numerical calculation.^24^ Multiple layers with specified thicknesses, defect densities, doping concentrations, and other physical characteristics are incorporated into this program to construct and model solar cells. It employs fundamental semiconductor formulas, including the Poisson and continuity equations for charge carriers. Numerous elements, including the distribution of the electric field (*E*), current density, transportation characteristics, generation, and recombination dynamics, can be described using these equations. The equations are written as follows:^25^1
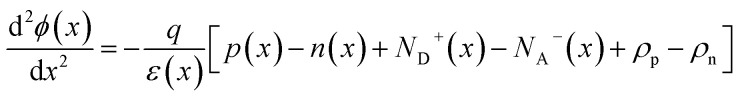
2
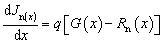
3
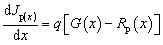


The drift-diffusion concept, which takes into account both electric field-driven and concentration gradient-driven carrier motion, provides a realistic description of the current transport that occurs in semiconductors. The total current densities for electrons (*J*_n_) and holes (*J*_p_) are given by:^26^4
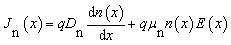
5
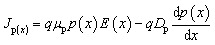
where *ψ* stands for electric potential, *q* is the elementary charge, and *ε* is the dielectric permittivity. The p and n are density of free electrons and holes, respectively. The *N*_D_^+^ and *N*_A_^−^ denote the ionized density of the donor and acceptor, respectively. The *ρ*_n_ and *ρ*_p_ are the density of trapped electrons and holes. The *J*_n_ and *J*_p_ represent total current density for electrons and holes. Finally, *G* (*x*) and *R* (*x*) are generation and recombination rates.

SCAPS-1D models recombination through Shockley–Read–Hall (SRH), radiative, and Auger mechanisms. SRH is dominant and depends on trap density and energy levels, while radiative and Auger recombination are considered in materials with high carrier densities or direct bandgaps.

6 *R*_rad_ = *B* (*n*_p_ − *n*_*i*_^2^)

7 *R*_Auger_ = *C*_n_*n*(*n*_p_ − *n*_*i*_^2^) + *C*_p_*p*(*n*_p_ − *n*_*i*_^2^)

8
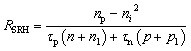
where *n*_*i*_ is Intrinsic carrier concentration. The *n*_1_ and *p*_1_ stand for carrier concentrations at trap energy level. The *τ*_n_ and *τ*_p_ denote carrier lifetime for electrons and holes, respectively. The *B*, *C*_n_, and *C*_p_ represent radiative and Auger recombination coefficients, respectively.

The physical parameters utilized in PV simulations were derived from previously reported studies.^23,27–30^ The physical characteristics of HTL, ETL, interfacial passivator, and absorber materials are listed in Table 1. Table 2 shows the key parameters at the interface between ETL/perovskite, perovskite/passivator, and passivator/HTL. All calculations were carried out with a 1.5 AM light spectrum, and the cell operating temperature was selected as 300 K. To enhance the efficiency of PSC, the *n*-Bu4S organic compound has been employed as a passivation interlayer between mixed-cation FA_0.97_MA_0.03_PbI_2.91_Br_0.09_ perovskite and Spiro-OMeTAD HTL.

**Table 1:** The input characteristics for all materials employed in the simulation

Parameter/unit	SnO_2_	FA_0.97_MA_0.03_PbI_2.91_Br_0.09_	*n*-Bu4S	Spiro-OMeTAD
Thickness (nm)	100	400	50	150
*E*_g_ (eV)	4	1.57	3	2.8
*χ*_e_ (eV)	4.3	3.9	2.4	2.2
*ε*_r_	9	10	4.5	3
*N*_C_ (cm^−3^)	1 × 10^19^	1 × 10^19^	1 × 10^19^	1 × 10^17^
*N*_V_ (cm^−3^)	1 × 10^19^	1 × 10^17^	1.0 × 10^19^	1 × 10^17^
*µ*_e_ (cm^2^ V^−1^ s^−1^)	100	4.2 × 10^−3^	4.2 × 10^−2^	2 × 10^−2^
*µ*_h_ (cm^2^ V^−1^ s^−1^)	25	6.5 × 10^−3^	6.5 × 10^−2^	2 × 10^−2^
*N*_D_ (cm^−3^)	1 × 10^17^	1 × 10^15^	—	—
*N*_A_ (cm^−3^)	—	1 × 10^15^	1.0 × 10^17^	1 × 10^17^
Charge type	Neutral	Neutral	Neutral	Neutral
Grading *N*_T_	Uniform	Uniform	Uniform	Uniform
Energetic distribution	Single	Single	Single	Single
*N*_T_ (cm^−3^)	1 × 10^16^	1 × 10^14^	1 × 10^15^	1 × 10^15^

**Table 2:** Interface parameters of ITO/SnO_2_/FA_0.97_MA_0.03_PbI_2.91_Br_0.09_/*n*-Bu4S/Spiro-OMeTAD/Au structure

Parameters/interfaces	SnO_2_/FA_0.97_MA_0.03_PbI_2.91_Br_0.09_	FA_0.97_MA_0.03_PbI_2.91_Br_0.09_/*n*-Bu4S	*n*-Bu4S/Spiro-OMeTAD
Defect type	Neutral	Neutral	Neutral
Capture cross section for electrons (cm^2^)	1 × 10^−19^	1 × 10^−19^	1 × 10^−19^
Capture cross section for holes (cm^2^)	1 × 10^−19^	1 × 10^−19^	1 × 10^−19^
Energetic distribution	Single	Single	Single
Reference for defect energy level *E*_t_	Above the highest *E*_v_	Above the highest *E*_v_	Above the highest *E*_v_
Energy with respect to reference (eV)	0.600	0.600	0.600
Total density (cm^−2^)	1 × 10^11^	1 × 10^11^	1 × 10^11^

## 3. Results and discussion

**Fig. 1:**
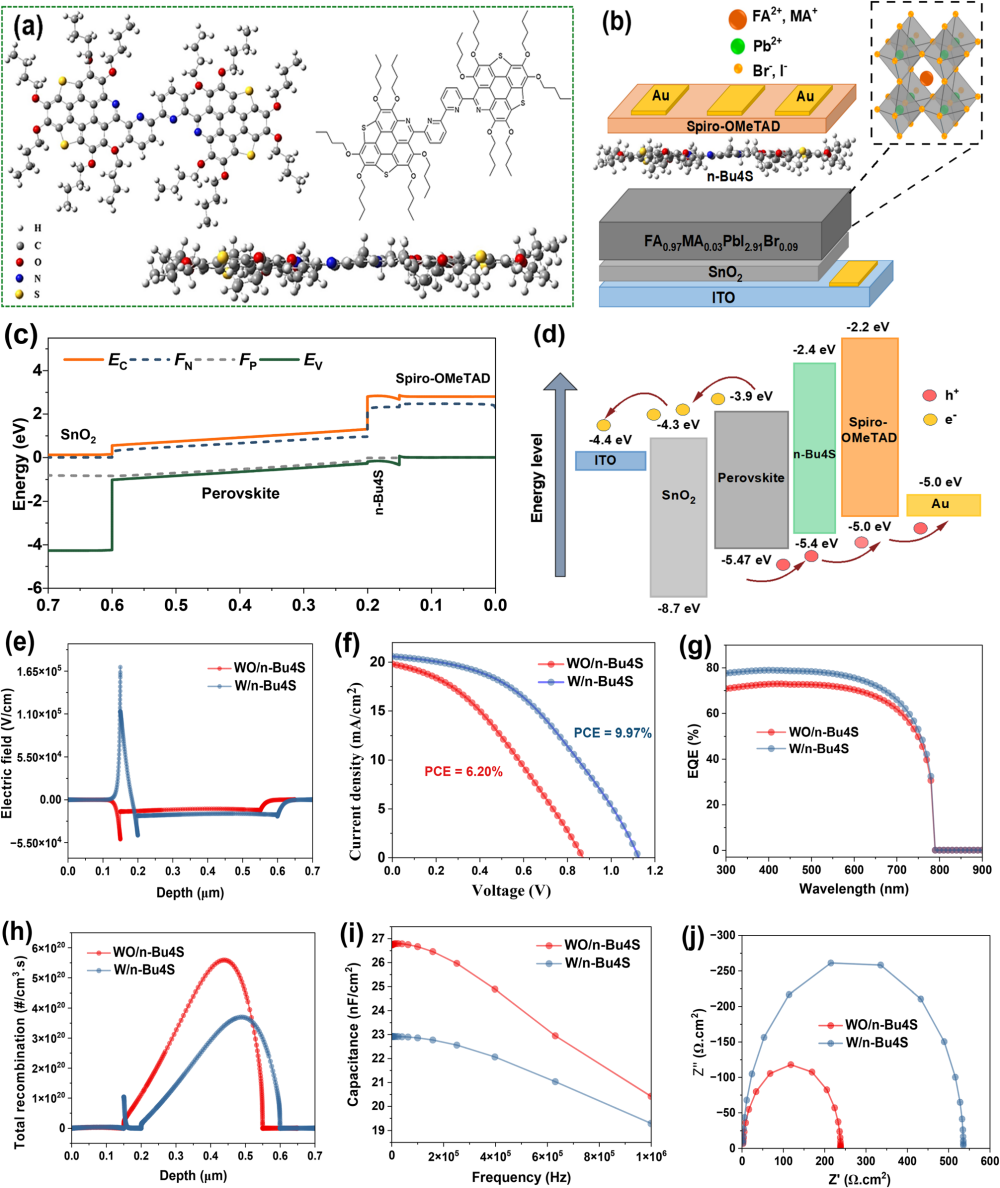
Schematic representation of the *n*-Bu4S molecule and initial simulation of PSCs. (a) Chemical structure of *n*-Bu4S compound designed by Gaussian 9 program. (b) The architecture of designed PSC. (c) Energy band diagram of simulated SnO_2_/FA_0.97_MA_0.03_PbI_2.91_Br_0.09_/*n*-Bu4S/Spiro-OMeTAD. (d) Energy level frontier for PSC materials. (e) Electric filed distribution. (f) The simulated *J*–*V* characteristics of the devices with and without *n*-Bu4S interlayer. (g) The corresponding EQE spectra. (h) Total recombination profiles of simulated devices. (i) *C*–*f* curves of PSCs with and without *n*-Bu4S treatment. (j) The calculated Nyquist plots. *Z*′ and *Z*″ are the real and imaginary parts of impedance spectrum.

The geometry optimizations of the designed *n*-Bu4S compound were investigated by density functional theory utilizing the Gaussian 09 program. The program used Becke's three-parameter hybrid function integrated with the Lee–Yang–Parr correlation function (B3LYP) and the 6-311++G** basis set in a vacuum. As depicted in Fig. 1a, a Lewis base molecule was proposed by integrating two chalcogen-containing moieties, bridged *via* a tetra-pyridine ligand. The constructed *n*-Bu4S interlayer is intended to increase molecules interaction with defects, hence enhancing defect passivation. Particularly, all four-guest chalcogen groups are strategically arranged to encapsulate under-coordinated Pb^2+^ ions and iodine vacancy defective centers, resulting in beneficial host–guest interactions. The existence of four additional pyridine ligands in the bridging unit further augments passivation by targeting both deep-level traps and halide vacancy defects. Furthermore, the insertion of several alkyl units at the molecular termini increased solubility on the perovskite surface while also imparting hydrophobicity to the perovskite film, resulting in improved PSC durability.^31^ The thiophene functionalities have negative charges, implying that the lone pair electrons in these units can neutralize the Pb^2+^/iodine vacancies within perovskite by Lewis acid-base interaction.^32^ Future work should include density functional theory calculations and experimental validation to confirm the proposed mechanism.

This work used a planar n-i-p PSC for device simulation. The cell framework consists of ITO/SnO_2_/FA_0.97_MA_0.03_PbI_2.91_Br_0.09_/*n*-Bu4S/Spiro-OMeTAD/Au as depicted in Fig. 1b. The corresponding energy band diagram (EBD) and charge transfer pathways for PSC are exhibited in Fig. 1c and d, respectively. Minimizing energy barriers by the smooth alignment of the conduction and valence bands across the *n*-Bu4S interlayer facilitates enabling effective carrier extraction and transport. Notably, the small conduction band offset between perovskite and ETL avoids spike formation, ensuring unimpeded electron flow.^33^ Simultaneously, favorable valence band alignment at the perovskite/*n*-Bu4S and *n*-Bu4S/HTL junctions drives effective hole extraction. The simulated EBD also affirms the expanded quasi-Fermi level splitting under light conditions, reflecting a high built-in potential (*V*_bi_) and thus confirming a higher *V*_OC_ and overall increased PV performance. This trend is further supported by the *E* distribution shown in Fig. 1e. In this *E*-field profile, a higher *E* demonstrates a stronger internal driving force acting on the photocarriers within the PSC. A strong *E* promotes separating the photo-induced excitons, decreasing their chances of recombination. The enhanced electric field near the interface indicates improved *V*_bi_, which facilitates efficient charge separation and reduces carrier recombination. This indicates stronger *V*_bi_ owing to better band alignment and possibly more efficient interface passivation by the *n*-Bu4S interlayer.

Fig. 1f shows simulated *J*–*V* curves of n-i-p PSCs with and without the *n*-Bu4S interfacial layer. The insertion of the *n*-Bu4S layer significantly improves the PV properties of PSC by increasing *J*_SC_, *V*_OC_, and FF. The photogenerated *J*_SC_ is increased from 19.80 to 20.59 mA cm^−2^ after incorporating *n*-Bu4S. The enhanced charge carrier generation is further confirmed by the EQE spectrum (see Fig. 1g). As observed, PSC treated with *n*-Bu4S showed a higher EQE response, reaching nearly 80%, compared to the untreated device, indicating enhanced light harvesting and carrier collection.^34^ This increment is attributed to the improved *V*_bi_, resulting in more effective separation of photocarriers. Besides, the favorable band alignment promotes smoother carrier transport due to suppressed interfacial losses. The *V*_OC_ value is significantly improved from 0.870 to 1.127 V for the passivated device. This enhancement can be explained by calculating the generation–recombination profile of PSCs. As shown in Fig. 1h, the *n*-Bu4S-passivated device exhibited a lower recombination rate. The chalcogen-containing groups and pyridine ligands in *n*-Bu4S coordinate with under-coordinated Pb^2+^ and iodine vacancies, passivating deep-level defects, which act as recombination sites. Passivation reduces non-radiative recombination, enabling higher splitting of electron and hole quasi-Fermi levels, directly contributing to higher *V*_OC_. Fig. 1i shows simulated *C*–*f* characteristic curves of PSCs measured at a frequency range of 10^2^ to 1 MHz. Lower capacitance at low frequencies of *n*-Bu4S-passivated PSC suggests reduced interfacial traps and mitigated ion migration or accumulation of mobile charges. Additionally, the calculated Nyquist plots show impedance spectra with only a single arc (Fig. 1j). The PSC with *n*-Bu4S showed a larger arc diameter. A larger arc indicates higher recombination resistance (*R*_rec_).^35^ Generally take place because of enhanced surface passivation and a suppressed recombination process at the interface, resulting in the higher FF of the *n*-Bu4S device.

**Fig. 2:**
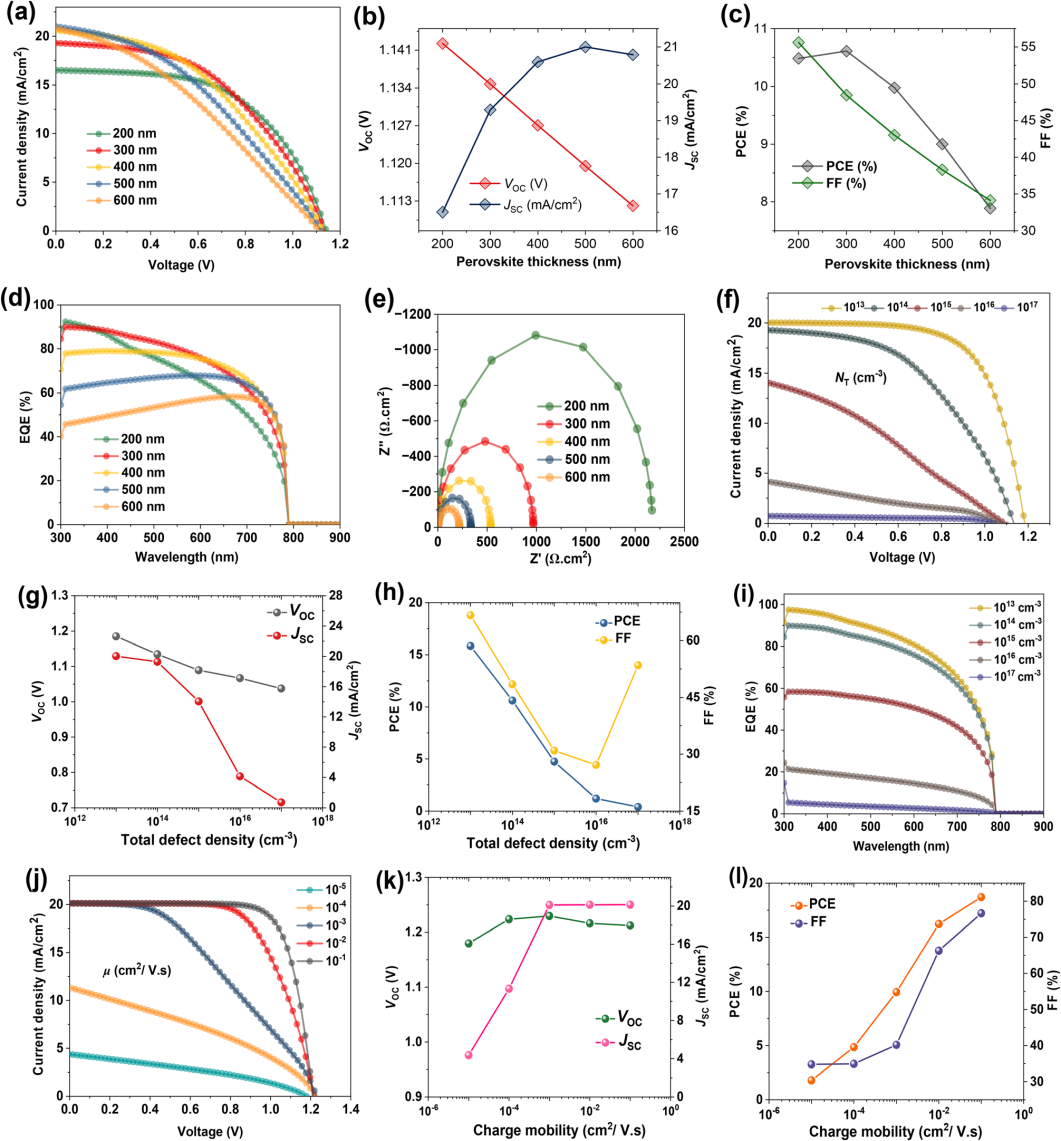
Optimization of perovskite absorber layer of *n*-Bu4S-treated PSCs. (a) *J*–*V* curves of the PSCs at different perovskite thicknesses. The related parameters includes: (b) *V*_OC_, *J*_SC_, (c) FF, PCE, (d) The calculated EQE, and (e) The simulated Nyquist plots. (f) *J*–*V* plots of devices with varying bulk defect densities of perovskite. Variation of (g) *V*_OC_, *J*_SC_, (h) FF, PCE, and (i) EQE. (j) *J*–*V* plots of the PSCs at different perovskite charge mobilities. Key parameters (k) *V*_OC_, *J*_SC_, (l) FF, and PCE.

As Fig. 2a illustrates, the PV properties of *n*-Bu4S-based devices clearly depend on the perovskite film thickness. As the thickness increases, the *J*_SC_ initially improves due to enhanced light harvesting, peaking at 300 nm (Fig. 2b). However, higher thickness results in lower FF, probably because of rising *R*_S_ and carrier recombination losses (Fig. 2c). The *V*_OC_ remains relatively stable, implying that the energetic alignment and interfacial features are maintained.^36^ The EQE spectra displayed in Fig. 2d reflect this tendency, where the 300 nm cell yields the highest photoresponse across the visible spectrum. Nyquist plots further confirm this, with the large *R*_rec_ revealed for the 300 nm-thick perovskite, indicating enhanced charge extraction and minimized non-radiative losses (Fig. 2e). Altogether, these findings highlight 300 nm as the optimal perovskite thickness for balancing light absorption and carrier collection, achieving a PCE of 10.61%.

The influence of varying bulk defect density (*N*_T_) in the perovskite film was studied, and the corresponding *J*–*V* curves are exhibited in Fig. 2f. The simulations demonstrate a pronounced reduction in PV parameters with increasing *N*_T_ value (Fig. 2g). This trend is attributed to increased non-radiative recombination and impaired carrier transfer caused by the increased presence of deep-level traps within the perovskite layer, which hinder efficient charge collection and decline overall PSC performance.^37^ As shown in Fig. 2h, the PCE is reduced from 15.83% to 0.39% after increasing *N*_T_ content from 10^13^ cm^−3^ to 10^17^ cm^−3^. This pattern is supported by EQE measurements (see Fig. 2i), which show a considerable decline in spectral response throughout the entire visible range for higher *N*_T_. The lower EQE indicates poor charge extraction efficacy owing to higher trap-assisted recombination, particularly in the high-energy region where absorbance is typically maximum. The optimized *N*_T_ was fixed at 10^13^ cm^−3^, corresponding to a charge diffusion length (*L*_n,p_) on the order of 300–400 nm, which is consistent with reported values for the mixed-cation lead mixed-halide perovskite films.^38^ Liang *et al.* reported carrier diffusion lengths for electrons and holes in the range of 320–660 nm depending on composition for MA/FA mixed perovskites,^39^ which is corroborated well with our SCAPS-1D calculations.

Fig. 2j illustrates variations of charge mobility (*µ*) in the perovskite film for *n*-Bu4S-based PSCs, which show a positive correlation with key PV characteristics. The *µ* value was varied from 10^−5^ to 10^−1^ cm^2^ V^−1^ s^−1^ within experimentally measured ranges for halide perovskites to ensure actual charge dynamics. Lim *et al.* reported high *µ* values for polycrystalline films mixed organic–inorganic perovskites in the range of 0.3 to 6.7 cm^2^ V^−1^ s^−1^.^40^ As *µ* increased, enhancements in FF, *J*_SC_, and PCE (Fig. 2k) suggested more effective charge transfer and suppressed recombination rates. Additionally, the *V*_OC_ remains relatively constant across the *µ* range, indicating that *µ* mainly influences charge generation rather than affecting the quasi-Fermi level splitting. As depicted in Fig. 2l, the PCE boosted to 18.7% with increasing *µ*, assigning better charge transport kinetics within the perovskite. Higher *µ* results in a longer *L*_n,p_, allowing charge carriers to move longer distances before recombining.

**Fig. 3:**
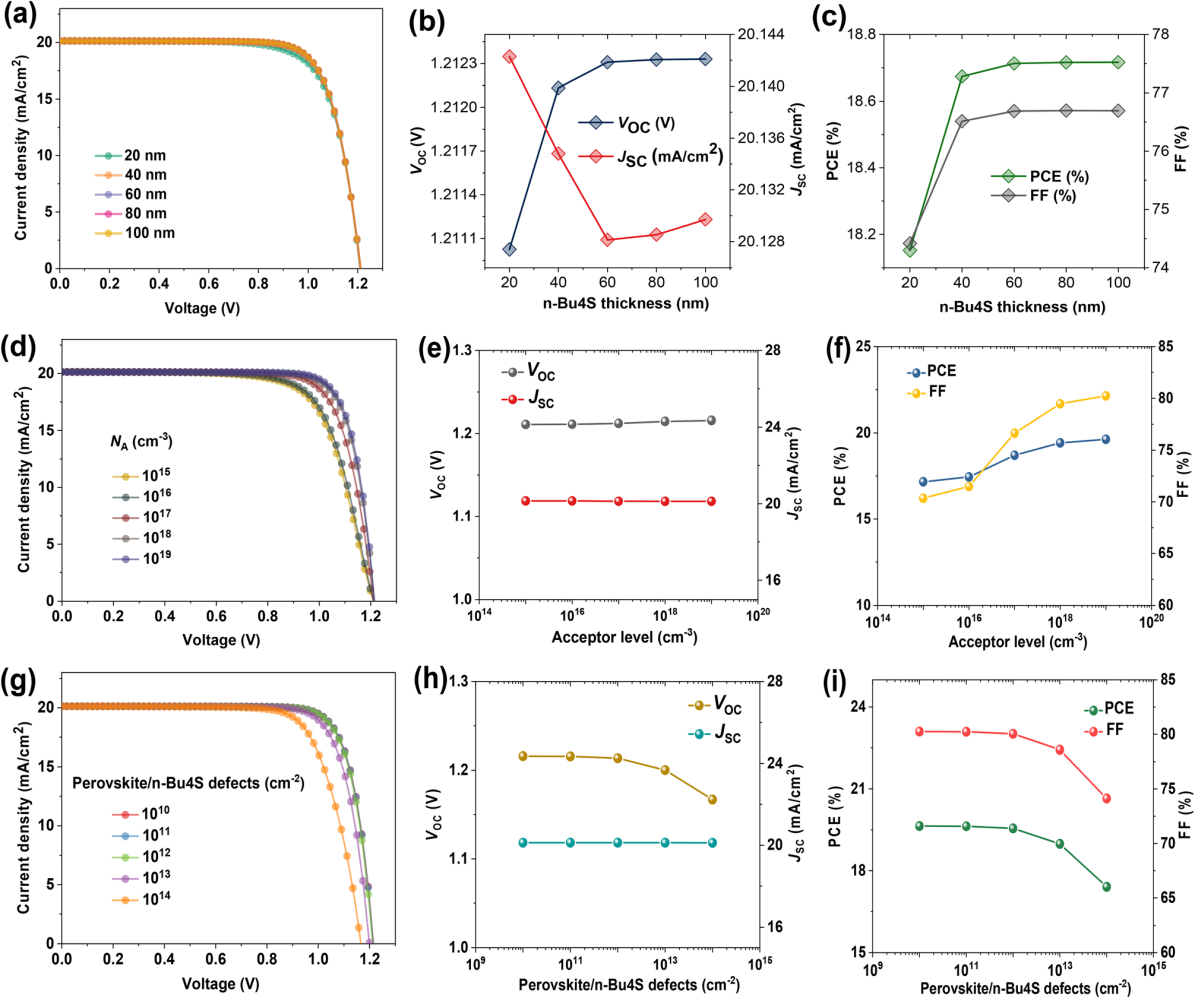
Optimization of *n*-Bu4S passivation layer and perovskite/*n*-Bu4S interfacial contact. (a) *J*–*V* plots of PSCs at different *n*-Bu4S thicknesses. The related parameters includes (b) *V*_OC_, *J*_SC_, (c) FF, and PCE. (d) *J*–*V* characteristics of PSCs with varying shallow acceptor concentration (*N*_A_) of *n*-Bu4S interlayer. The variations in (e) *V*_OC_, *J*_SC_, and (f) FF, PCE. (g) *J*–*V* plots of PSCs with changing defect density at perovskite/*n*-Bu4S interface. The corresponding PV parameters (h) *V*_OC_, *J*_SC_, and (i) FF, PCE.

To further probe the effect of interfacial passivation on PSCs, we varied the thickness of the *n*-Bu4S interlayer in a range of 20–100 nm, as shown in Fig. 3a. The *V*_OC_ and *J*_SC_ are almost constant with increasing *n*-Bu4S thickness (Fig. 3b). Whereas, a moderate increase in *n*-Bu4S thickness leads to a slight enhancement in both FF and efficiency, which can be ascribed to improved surface passivation that reduces charge recombination at the interface (Fig. 3c). An increase in *n*-Bu4S spacer thickness showed minor enhancement in PCE from 18.15% to 18.71%, implying its main role as interfacial modification rather than bulk charge transport. More significantly, a clear increase in FF and PCE was obtained by raising the shallow acceptor doping concentration (*N*_A_) in the *n*-Bu4S interlayer, as shown in Fig. 3d–f. This is mostly related to better charge transfer and extraction, as higher doping levels generate a stronger *V*_bi_ and enhance the electrical conductivity, therefore enabling effective carrier collection.^41^ On the other hand, Fig. 3g–i reveal that increasing the defect traps at the perovskite/*n*-Bu4S contact caused performance reductions. Specifically, a reduction in *V*_OC_, FF, and PCE was observed, indicating that under such conditions, trap-assisted non-radiative recombination processes predominate. Following the optimization of the *n*-Bu4S interfacial layer, the device achieved a PCE of 19.63%, underscoring the pivotal role of interfacial quality in governing the overall performance of PSCs.

**Fig. 4:**
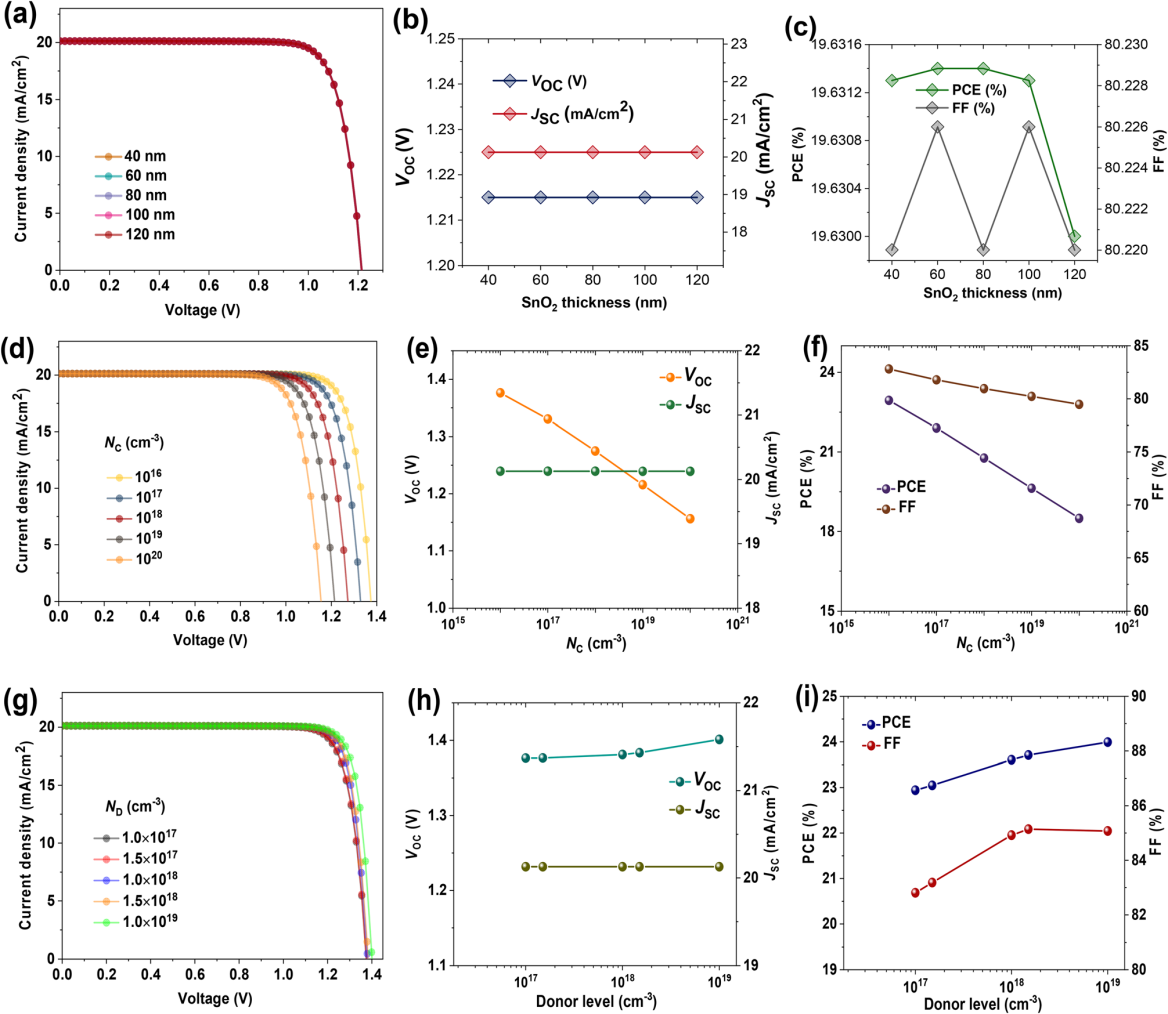
Optimization of SnO_2_ ETL material of PSCs with *n*-Bu4S treatment. (a) *J*–*V* plots of PSCs at different SnO_2_ thicknesses. The related parameters includes (b) *V*_OC_, *J*_SC_, (c) FF, and PCE. (d) *J*–*V* characteristics of PSCs *versus* effective density of states at conduction band (*N*_C_) of SnO_2_ layer. The variations in (e) *V*_OC_, *J*_SC_, and (f) FF, PCE. (g) *J*–*V* plots of devices with changing shallow donor concentration (*N*_D_) of SnO_2_ layer. The corresponding PV parameters (h) *V*_OC_, *J*_SC_, and (i) FF, PCE.

The performance of PSCs with *n*-Bu4S treatment was further optimized by adjusting the properties of the SnO_2_ ETL. Fig. 4a demonstrates the simulated *J*–*V* characteristics of the devices calculated at different SnO_2_ thicknesses. Changing the thickness of the SnO_2_ film had no effect on cell efficiency, implying that within the investigated range, carrier extraction and optical losses had no appreciable influence (Fig. 4b and c). By contrast, a notable reduction in *V*_OC_, FF, and PCE was observed due to increasing the effective density of states in SnO_2_'s conduction band (*N*_C_), as illustrated in Fig. 4d. This improvement is associated with decreased recombination processes due to lower electron populations in the conduction band, which can enhance band alignment and charge selectivity.^42^ The device with 10^16^ cm^−3^*N*_C_ obtained a champion PCE of 22.94% with a *V*_OC_ of 1.37 eV (Fig. 4e and f), assuming a balance between charge carrier mobility and extraction. The reported high *V*_OC_ represents an upper-bound scenario, and realistic device performance is expected at higher *N*_C_ values. On the other hand, as shown in Fig. 4g, increasing the donor doping level (*N*_D_) in SnO_2_ led to enhancements of *J*–*V* metrics. Higher *N*_D_ enhances the electrical conductivity of the SnO_2_ and strengthens the *V*_bi_ field at the SnO_2_/perovskite interface. This promotes faster carrier extraction and suppresses charge recombination. The best-performing PSC of 24% was attained at an *N*_D_ of 10^19^ cm^−3^ (Fig. 4h and i), emphasizing the significance of both electronic tuning and doping strategies in optimizing SnO_2_ design.

**Fig. 5:**
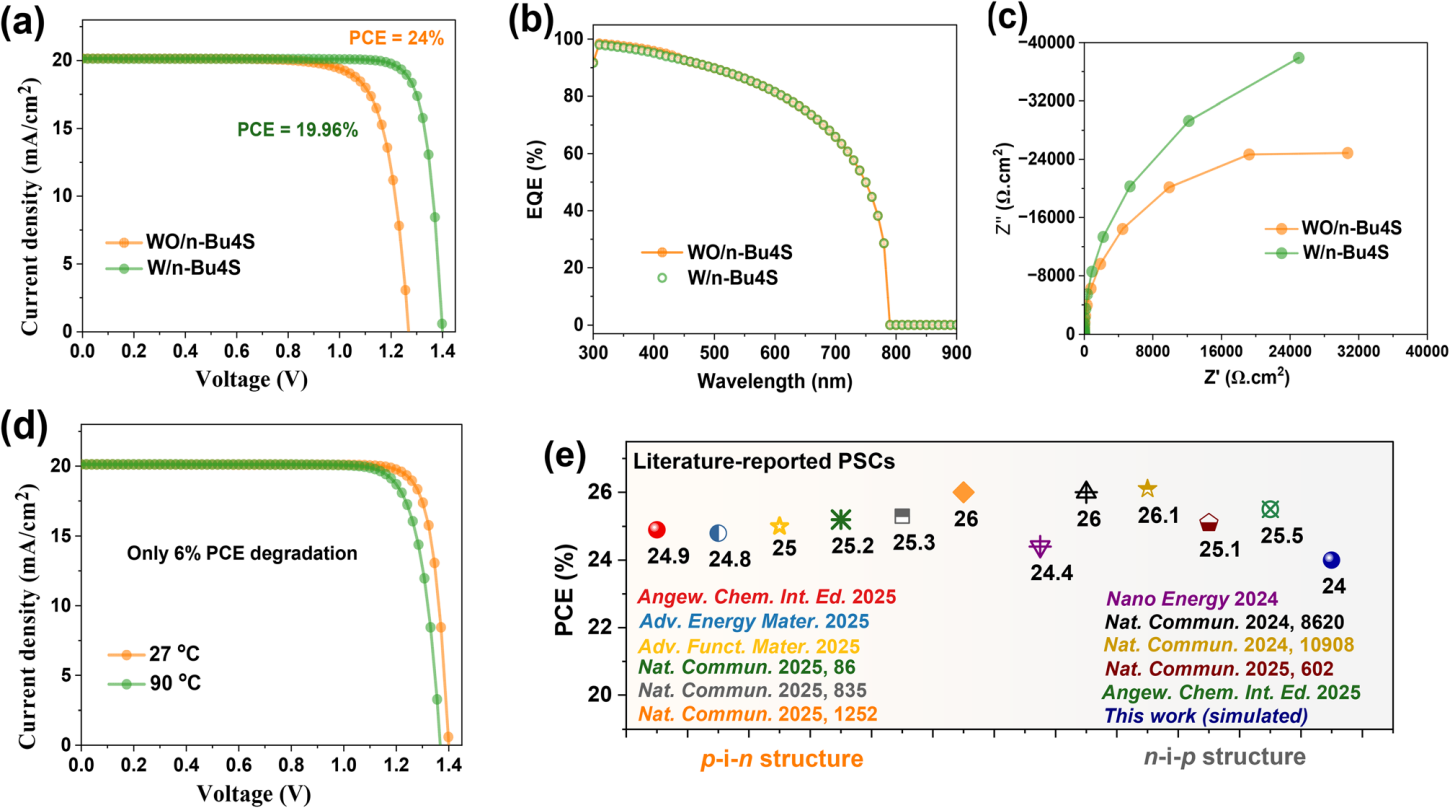
Comparison of the final optimized PSCs with and without the *n*-Bu4S interfacial engineering, alongside previously reported studies. (a) *J*–*V* curves of optimized PSCs with and without *n*-Bu4S interlayer. (b) EQE profiles of PSCs. (c) Nyquist plots. (d) Thermal stability for the *n*-Bu4S-treated PSCs at 27 °C and 90 °C. (e) Partial summary of the performances for the state-of-the-art rigid PSCs with interfacial passivation reported in the literature.^23,43–52^

Fig. 5a shows a comparative analysis of the *J*–*V* of PSCs without and with *n*-Bu4S interfacial engineering. The PV calculations demonstrate a remarkable increase in FF, *V*_OC_ and PCE for the cell with the *n*-Bu4S interlayer, achieving a performance of 24% compared to 19.96% for the untreated cell. However, the *J*_SC_ remains constant, suggesting that the bulk photo-generation of carriers in the perovskite film is unaffected by the *n*-Bu4S passivation. This behavior is further confirmed by the EQE profile (Fig. 5b), which reveals closely identical responses across the wavelength range for both cells. Furthermore, Nyquist curves obtained from impedance spectrum computations (Fig. 5c) show that the passivated PSC has a larger semicircle in the high-frequency range, thereby indicating greater *R*_rec_. This suggests that the effective reduction of non-radiative recombination at the interfaces by the *n*-Bu4S interlayer facilitates the improvement of the general performance. The insertion of the *n*-Bu4S interlayer efficiently reduces carrier recombination, particularly at the perovskite/transport layer interfaces. The enhanced *R*_rec_ is attributed to reduced trap-state density and minimized defect-mediated (SRH) recombination pathways, which leads to enhanced carrier lifetime and more efficient charge extraction. The suppression of recombination is consistent with the observed improvement in *V*_OC_ and FF, confirming that interfacial defect passivation is the dominant mechanism enhancing PSC efficiency. In the absence of an *n*-Bu4S interlayer, carrier transport across the perovskite/spiro-OMeTAD interface is often restricted by defect states and energy level mismatch, which can lead to carrier trapping.

The temperature-dependent performance of the optimized *n*-Bu4S-based PSC was evaluated using SCAPS-1D by simulating the *J*–*V* scans at 300 K (27 °C) and 363 K (90 °C) under AM1.5 G illumination. In the simulation, only the operating temperature was varied, while all other device parameters were kept constant. Fig. 5d illustrates the *J*–*V* curves of the optimized devices at room and high temperatures. The reduction in efficiency from 24% to 22.58% at elevated temperatures is mainly attributed to the decrease in *V*_OC_ and FF caused by increased carrier recombination. The performance degraded by approximately only 6% when the temperature increased from 27 to 90 °C, underscoring the thermal stability of PSCs with *n*-Bu4S engineering. The thermally robust performance can be ascribed to the multifunctional role of the *n*-Bu4S interfacial passivation. The designed molecular bridging passivates under-coordinated Pb^2+^ ions and iodine vacancies through strong Lewis acid–base bonding by chalcogen and pyridine units, suppressing thermally activated non-radiative recombination at high temperatures. The minimal decrease in *J*_SC_ and FF at 90 °C suggests maintained carrier transport and limited ion migration.

To evaluate the effectiveness of our simulated PSC, we compared the PCE of our designed PSC with previously published devices. Fig. 5e displays a comparative diagram for the state-of-the-art PSCs reported in the literature. As exhibited in the diagram, our PSC shows competitive performance, positioning among the top-performing PSCs. Importantly, the simulated PCE of 24% falls within the range of experimentally reported efficient PSCs, indicating realistic parameter selection; however, these results represent idealized device conditions. This emphasizes how successfully our interfacial engineering approach and material optimization improve device stability and efficiency.

Table 3 summarizes the progressive enhancement of *n*-Bu4S-based PSC with each optimization step. Starting from a reference PSC, modifications were sequentially introduced, including (i) incorporation of the *n*-Bu4S interlayer, (ii) defect density optimization, and (iii) thickness optimization.

**Table 3:** Step-by-step optimization workflow and corresponding PV parameters of the simulated PSCs

Step	Layer	Parameter	Value	Optimized	*V*_OC_ (V)	*J*_SC_ (mA cm^−2^)	FF (%)	PCE (%)
1	Baseline device	Reference structure	—	—	0.87	19.80	35.97	6.20
2	Interfacial modification	*n*-Bu4S interlayer	—	—	1.12	20.59	43.00	9.97
3	Perovskite absorber	Thickness	200–600 nm	300 nm	1.13	19.29	48.46	10.61
		Defect density	10^13^–10^17^ cm^−3^	10^13^ cm^−3^	1.18	20.04	20.04	15.83
		Charge mobility	10^−5^–10^−1^ cm^2^ V^−1^ s^−1^	10^−1^ cm^2^ V^−1^ s^−1^	1.21	20.12	76.65	18.70
4	*n*-Bu4S interlayer	Thickness	20–100 nm	40 nm	1.21	20.14	76.51	18.67
		Acceptor doping	10^15^–10^19^ cm^−3^	10^19^ cm^−3^	1.21	20.12	80.22	19.63
5	Perovskite/*n*-Bu4S interface	Defect density	10^10^–10^14^ cm^−3^	10^10^ cm^−3^	1.21	20.12	80.24	19.64
6	SnO_2_ ETL	Thickness	40–120 nm	60 nm	1.21	20.12	80.24	19.64
		Conduction density of states	10^16^–10^20^ cm^−3^	10^16^ cm^−3^	1.37	20.13	82.81	22.94
		Donor doping	10^17^–10^19^ cm^−3^	10^19^ cm^−3^	1.40	20.13	85.07	24.00

## 4. Conclusion

In conclusion, we reported a Lewis base molecule consisting of a thiophene ligand (*n*-Bu4S) bridged *via* a tetra-pyridine unit for passivating under-coordinated Pb^2+^ and iodine vacancy traps due to strong host–guest interaction. Then, a numerical simulation was performed on PSCs with and without an *n*-Bu4S passivation interlayer using the SCAPS-1D tool. Initially, increasing the thickness of the perovskite absorber enhanced light harvesting and *J*_SC_; yet, too thick layers caused reduced FF owing to increased *R*_S_ and insufficient charge extraction. Likewise, bulk defects in the perovskite and at the perovskite/*n*-Bu4S contact were shown to be important in carrier recombination; larger defect densities greatly lowered *V*_OC_, FF, and PCE. Moreover, enhancing the charge carrier mobility inside the perovskite raised the diffusion length and carrier lifetime, improving the PV characteristics and charge-collecting efficiency. By adding *n*-Bu4S, device performance improved significantly, and a PCE of 24% was obtained together with improved thermal stability. The beneficial defect passivation at the perovskite interface allows lowered interfacial recombination losses, which is mostly responsible for the high efficiency. This finding was further supported by Nyquist analysis, which exhibited increased recombination resistance in *n*-Bu4S-treated devices compared to their untreated counterparts.

## Conflicts of interest

The authors declare no conflict of interest.

## Data Availability

The data will be available from the corresponding author on reasonable request.
